# Bedside rounds in the hospital environment from the perspective of multiprofessional health teams

**DOI:** 10.1590/0034-7167-2023-0493

**Published:** 2024-11-22

**Authors:** Tauana Wazir Mattar e Silva, Marília Alves, Isabela Silva Câncio Velloso, Carolina da Silva Caram, Kateryna Metersky, Rita de Cássia Oliveira

**Affiliations:** I Centro Universitário Newton Paiva. Belo Horizonte, Minas Gerais, Brazil; II Universidade Federal de Minas Gerais. Belo Horizonte, Minas Gerais, Brazil; III Toronto Metropolitan University. Toronto, Canada

**Keywords:** Interprofessional Relations, Hospitals, Patient Care Team, Professional Practice, Health Knowledge, Attitudes, Practice, Relações Interprofissionais, Hospitais, Equipe de Assistência Multidisciplinar, Prática Profissional, Conhecimentos, Atitudes e Prática em Saúde, Relaciones Interprofesionales, Hospitales, Grupo de Atención al Paciente, Práctica Profesional, Conocimientos, Actitudes y Práctica en Salud

## Abstract

**Objective:**

To analyze the configuration of power relations among the multiprofessional team in the bedside round process in the hospital.

**Methods:**

Qualitative research with data analyzed through discourse analysis, based on Michel Foucault’s theoretical framework. From September to December 2022, we conducted interviews and field observations with the multiprofessional team at a hospital in Belo Horizonte, Minas Gerais, Brazil, as well as qualitative, semi-structured interviews with 37 professionals.

**Results:**

The participants pointed out that the experiences of the professionals involved in bedside rounds depend on how the physician conducts the process, and the physician-centered process makes it difficult for other professionals in the team to participate.

**Final considerations:**

The way hospitals organize bedside rounds does not promote knowledge articulation for their professionals. It hinders the circulation of power and harms interdisciplinary work in a process that maintains the physician as the main actor in clinical decisions.

## RESUMO

**Objetivo:**

Analisar a configuração das relações de poder entre a equipe multiprofissional no processo de ronda a beira leito hospitalar.

**Métodos:**

Pesquisa qualitativa com dados analisados por meio de análise de discurso, com base no referencial teórico de Michel Foucault. De setembro a dezembro de 2022, realizamos entrevistas e observações de campo com a equipe multiprofissional de um hospital em Belo Horizonte, Minas Gerais, Brasil, e entrevistas qualitativas semiestruturadas com 37 profissionais.

**Resultados:**

Os participantes relataram que as experiências dos profissionais envolvidos nas rondas à beira do leito dependem da forma como o médico conduz o processo, e o processo centrado no médico dificulta a participação dos demais profissionais da equipe.

**Considerações finais:**

A forma como os hospitais organizam as rondas à beira do leito não promove a articulação do conhecimento de seus profissionais. Além disso, ela dificulta a circulação de poder e prejudica o trabalho interdisciplinar em um processo que mantém o médico como o principal ator nas decisões clínicas.

## RESUMEN

**Objetivo:**

Analizar la configuración de las relaciones de poder entre el equipo multiprofesional en el proceso de rondas de cabecera en el hospital.

**Método:**

Investigación cualitativa con datos analizados a través del análisis del discurso, basado en el marco teórico de Michel Foucault. De septiembre a diciembre de 2022, realizamos entrevistas y observaciones de campo con el equipo multiprofesional de un hospital de Belo Horizonte, Minas Gerais, Brasil, así como entrevistas cualitativas semiestructuradas con 37 profesionales.

**Resultados:**

Los participantes señalaron que las experiencias de los profesionales involucrados en las rondas de cabecera dependen de cómo el médico conduce el proceso, y el proceso centrado en el médico dificulta la participación de otros profesionales del equipo.

**Consideraciones finales:**

La forma en que los hospitales organizan las rondas de cabecera no promueve la articulación del conocimiento de sus profesionales. Además, dificulta la circulación del poder y perjudica el trabajo interdisciplinario en un proceso que mantiene al médico como actor principal de las decisiones clínicas.

## INTRODUCTION

Health care provision requires cooperation among many different professionals. It requires effective communication mechanisms capable of guaranteeing collaborative and safe care^(1)^. Bedside rounds are one such communication tool that allows health team professionals to share and deepen knowledge in the context of health work^(2)^. It involves interaction between different professionals and discussion of the contributions of each member of the team in the therapeutic organization in an integrated way^(3)^. Thus, it is a process that provides a good opportunity to perform an efficient anamnesis and physical assessment, thereby developing effective and patient-centered communication^(4)^.

Despite being a valuable care planning tool, few studies explore the configuration of bedside rounds and the integration of health professionals in this process. Most of the published studies analyze patients’ perceptions and feelings about bedside medical visits^(5)^. Others focus on the role of the physician in the rounds, their perceptions regarding bedside rounds as a strategy for training new doctors, the impact of this process on expanding medical knowledge, and on collaborative information systems in rounds for communication between physicians^(6-8)^. The wider hospital environment in which rounds occur and the modes of participation amongst multiprofessional team members carrying out rounds in this context are areas that have received little academic attention.

Given this gap, the present study focuses on the configuration of bedside rounds in a Brazilian hospital’s inpatient unit (IU) and intensive care unit (ICU). We use Michel Foucault’s conception of power-knowledge relations to develop our analysis. Foucault suggested that everyday practices constitute power-knowledge relations, in which the subject’s knowledge determines their position in social structures within an established relationship. Thus, in the professional relationships of the health team, those with greater knowledge assume a privileged position in power relations, considering that discourses of knowledge legitimize power^(9)^.

Foucault’s thought centers on the correlation between power and knowledge, so that knowledge is related to the truth legitimized through discourse^(10,11)^. Between knowledge techniques and power strategies, there is a field of force correlations, in which one must analyze the power relations^(12)^.

According to Foucault’s perspective, everyday relationships and practices constitute power. The environment, a social space of construction for the subject and a crucial tool in organizing this power, influences it^(10)^. The hospital is an environment that transforms the vision of power because the power exercised in it is conceived as a strategy that causes the domination of a professional over others, contributing to a network of tense relationships^(9)^.

In this context, the present study seeks to answer the following question: how are power relations among multiprofessional team professionals configured in the bedside rounds process? It is essential to understand the dynamics of bedside rounds, which requires considering the context’s influence on power exercise, the establishment and maintenance of professional boundaries, and the effects these professional relationships have on the subjects involved.

## OBJECTIVE

To analyze the configuration of power relations that permeate the multiprofessional team during the bedside round process.

## METHODS

### Ethical considerations

This study complied with the National Health Council’s Resolution Number 466/2012 and Resolution Number 510/2016. The Ethics Committees of the Federal University of Minas Gerais (UFMG) approved the project and the hospital for the study scenario. Before data collection, participants received information about the study and signed an informed consent form without any coercion. Finally, we obtained the Informed Consent Form in writing.

### Design and theoretical framework

This study aligns with the philosophical underpinnings of qualitative research; therefore, this was considered an appropriate method of inquiry. We chose the qualitative approach to enhance our understanding of daily life in this hospital setting and the experiences of individuals within it^(13)^.

Given these considerations, this qualitative research is based on Michel Foucault’s post-structuralist perspective. This study adopts post-structuralism as the theoretical outline, which manifests itself as an attempt to deconstruct concepts declared as absolute truths by considering reality as a subjective social construction. Thus, post-structuralism is a perspective that questions how society structures itself in a particular context and moment^(14)^.

This article followed the writing recommendations of the Consolidated Criteria for Reporting Qualitative Research (COREQ). It also preserved clarity in these three domains: research team and reflexivity (personal characteristics and relationship with participants), study design (theoretical framework; participant selection; setting, and data collection), and analysis and findings (data analysis and reporting).

### Methodological procedures

#### Study setting

We opted to study the IU and the ICU to better understand how different environments influence subjects’ experiences and perceptions^(9)^. IUs are sectors for hospitalizing of non-critical patients. Patient turnover is high in these units, and several professional specialties work within them simultaneously^(15)^. On the other hand, ICUs have a more limited, specialized professional team to provide care to critically ill patients who require complex care^(16)^.

At the time of data collection, the IU had 42 beds and a care team composed of six resident physicians, two daytime nurses, one night nurse, 16 nursing technicians, and an indeterminant number of mixed clinical staff. Thus, it was not possible to precisely define the number of medical professionals working in the IU. The ICU had 40 beds and a care team composed of three hospitalist physicians, 36 on-duty physicians, nine resident physicians, one in-house staff nurse, 17 duty nurses, and 100 nursing technicians.

#### Study participants

The sample included 11 physicians, 18 nurses, and eight key informants (physiotherapists, nursing technicians, and psychologists). One invited ICU physician refused to participate in the study. We selected the physicians and nurses using a non-probabilistic, convenience sample. The other participants were enrolled as key informants for two reasons: they were explicitly mentioned by the interviewees (nurses and physicians) in many moments; and they had shown outstanding behavior during the observation of the bedside rounds in either sector. We interviewed key informants through informal conversations. Such conversations serve as a starting point for deepening knowledge about the phenomenon under investigation because key informants are holders of strategic information^(17)^.

There was no a priori delimitation of the number of interviewees, and data saturation served as the criterion for defining the sample. When new data repeated what was expressed in previously collected data, interviews were discontinued^(18)^.

We selected participants based on the following eligibility criteria: resident physician; clinic coordinator physician or physician on duty; nurse under permanent contract in the hospital; and at least six months of experience in the units, either IU or ICU. We excluded professionals on vacation or sick leave during the data collection period.

#### Data collection and organization

Data collection occurred from September to December 2022 through systematic field observation and semi-structured interviews. The sequence of interviews was random, as we respected participants’ availability during their respective shifts. We conducted the interviews in a private location within the health unit, recorded them on a media player device, and later transcribed them in full to ensure the completeness and reliability of the information. Observational data were documented in a field diary.

Two experienced nurse researchers collected the data in addition to triangulating sources to ensure the study’s credibility and reduce the risk of data distortion.

We conducted a pilot study to validate the data collection instruments and assess the applicability of the observation plan and the semi-structured script. The study’s conclusions did not include the pilot test data. To elicit a discussion from the participants about the bedside round process in their respective sectors, we posed the question, “Tell me about the bedside round process in your work sector.”

To ensure the participants’ anonymity, they received a code using the initial capital letter corresponding to their professional category (physician - P; nurse - N; key informant - KI; physical therapist - PHY; nursing technician - NT; psychologist - PSY), followed by the number of the sequential order of the interview.

#### Data analysis

The analyzed data results from the observation executed on the sectors and of the semistructured interviews, whereas it wasn’t possible to perform a documental analysis as there were no documents regarding the bedside rounding process.

Michel Foucault’s theoretical framework, which enables understanding the dynamics of processes and forms of social production of meaning, served as the basis for the discourse analysis of the data^(10)^. Discourse analysis makes it possible to identify the characters, the lexical concept, and the nature of the vocabulary. In addition, it allows for selecting the ideas exposed and knowing the elements silenced by the participants through non-verbal means^(19)^.

Sorting, classification, and final analysis were the steps to data operationalization^(13)^. We used the MAXQDA® version 2022 software to code and organize our data based on the meanings extracted from the discourses. The data analysis led to the creation of a new category: professional discourses about bedside rounds.

### Rigour and reflexivity

Using rigor and reflexivity criteria is one of the ways to improve the quality and applicability of qualitative research. In this regard, it is crucial to provide a detailed description of the study so that one can better analyze its real dimension and the potential to use its findings^(20)^.

The methodological procedures, which included a detailed and thick description of the setting and participants, prolonged involvement of researchers in the field, persistent observation, triangulation of data, and peer review, established credibility. The possibility of applying the findings in other contexts, settings, or groups guarantees transferability, even though the intention is not to create sustainable generalizations in all scenarios. Therefore, a field diary provided a detailed description of the observation process. Finally, confirmability aims to ensure that the findings are logical^(21)^. We achieved this by gradually verifying strategies throughout the data collection process, allowing for necessary corrections, and conducting a peer review of the transcribed data.

Please note that this study is a component of ongoing doctoral research. Upon completion, we will present the findings to both the participants and the hospital managers.

## RESULTS

### Bedside rounds in the institutional setting

On our research site, bedside rounding is not an institutionally regulated practice. We observed that there were no documents to define guidelines and norms that standardize this practice, either in the IU or ICU. At the IU, physicians and other health professionals do not conduct rounds together. Health professionals assess the patients individually, at different times, and without multiprofessional communication and interaction. The medical team conducts rounds at the ICU in the morning, without a set time, with the participation of physiotherapy and nursing professionals.

In both the IU and ICU, physicians and nurses are exclusive professionals in these sectors; that is, they do not move between the IU and ICU. Professionals from other specialties, on the other hand, assist patients in each sector with on-demand care. In daily practices within the hospital, the physician establishes the monitoring and implementation of the daily care plan and oversees all essential processes in the conduct of clinical cases. This process is carried out by the doctor on duty and the rest of the multiprofessional team.

In this study, we observed that the team’s participation in bedside rounds depends on the profile and characteristics of the physician conducting it, which reinforces the physician’s centrality in the process.

### Professional discourses about bedside rounds

Of the 37 participants who comprised the study sample, most were nurses (45.9%). 67.6% of the sample identified as female, with 16 of these 25 women involved in nursing activities. In terms of age, 40.5% of the sample was between 20 and 25 years old. With respect to academic background, qualifications ranged from a technical level (two key informants) to graduation. Experience in the sector varied from one to five years (57.1%). Of the 19 participants who reported having two jobs, eight were physicians, nine were nurses, and two were physical therapists.

As for the particularities of the IU and ICU within this hospital, in general, we noted that, despite being unstructured, conflicts among professionals during bedside rounds are less evident in the ICU than in the IU. In the ICU, a physician mentions that the way bedside rounds happen has changed over time. The professional quoted below suggests that ICU rounds used to have a defined structure, but that doesn’t happen that way anymore. Currently, it tends to be faster.

*Formerly in ICU’s, we had structured bedside rounds, which were more complete and aimed at reviewing history, prescriptions, exam results, and... a little more time consuming, a little more complete.* (P_ICU_1)

For the convenience of the physician, rounds in the ICU typically take place in the morning, even though there is no set time for them to occur.

*And usually they* [physicians] *arrive around nine, between nine and ten o’clock, before the visit that is at ten thirty....* (N_ICU_4)

*The bedside round is basically done in the morning. At the latest, at the beginning of the afternoon, around noon.* (N_ICU_12)

*It is one bedside round per day, every day, always in the morning, but each unit has its bedside round at a time that is more... convenient....* (P_ICU_1)

The professionals who usually participate in the bedside round in the ICU are the physician coordinator, the physician on duty, residents, nurses, and a physiotherapist. As the following respondent noted, the presence of other professionals within the multiprofessional team is uncommon.

[the round] *is accompanied by the physician coordinator, the on-duty physician, usually the resident, or the residents who are taking care of that number of patients, the nurse, and the physiotherapist. We do not have a round with more professionals from other areas.* (N_ICU_3)

The ICU does not expect the nursing technician to participate in clinical discussions at the bedside. However, the excerpt below shows that despite not being a formal participant, this professional is available to contribute with any information the physician may need:

*Nursing technicians do not participate directly in the bedside rounds with doctors, but we are always close. ...if they need it, they ask.* (IC_TE_CTI_2)

The participants pointed out that the experiences of the professionals involved in bedside rounds depend on how the physician conducts the process.

*It depends on several factors because the bedside round is done by the physician coordinator. We currently have three coordinators, so each bedside round works in a unique way with each* [coordinator].... *Some are more detailed, ask broader questions. Others are more direct and more restricted to things much faster and more specific.* (P_ICU_3)

*If it is a round of one physician, it is okay! It is possible to fit right in. Depending on the other* [physician], *we can’t fit in... it is a very physician-centered thing. But there is another physician who is cool with it. It depends a lot on who is doing the bedside round.* (N_ICU_1)

In addition to the morning round that takes place in the presence of physicians to define clinical decisions, there is another moment of clinical discussion with the multiprofessional team in the ICU.

*I found out that there is another bedside round in the afternoon, in which the medical coordinator goes to the beds with the multiprofessional team and the residents. As far as I know, it’s a novelty. It’s not something frequent here*. (M_CTI_4)

*In the afternoon there is another bedside round. It is a different round from the morning one, and it is new. It has been happening for a short time*. (E_CTI_5)

Since it is not possible to carry out a single round with all the necessary professionals due to medical unavailability, it was necessary to create another round in the ICU.

The general way bedside rounds are conducted in the IU is not too different from the ICU. However, a participant noted that physicians in his unit perform bedside rounds individually, rather than as part of a larger team of health professionals. Additionally, the physician stated that the process prioritizes the physician’s needs over the patient’s.

*Oh, this process varies a lot from the team we are running, from the preceptors, right?! Each preceptor has a style, a different dynamic of running... so, it varies a lot from preceptor to preceptor .... The schedule also varies*. (P_IU_5)

*We used to have a multiprofessional bedside round, but our biggest difficulty here, our obstacle was always the physician’s participation, because in the IU there are several medical teams, and the coordinators of the medical teams do rounds on their own time. So, it wasn’t a multi-team bedside round. It was a doctor’s bedside round, and we tried to participate, but the schedule was very poorly established, and we couldn’t keep up.* (KI_PSY_1)

This physician-centered process makes it difficult for other professionals on the team to participate. There is a lack of integration between medical teams and other healthcare professionals.

*So, usually in the morning, the doctors* [residents] *have up to noon to go to the patient, and assess the patient, and reassess the patient too... And many times, we don’t see it. There are many doctors, a varied team... so they pass by, assess the patient, and leave.* (E_UNI_5)

*A multiprofessional bedside round? There isn’t one.* (N_IU_6)

*The teams are kind of independent, separated… Residents talk among residents. ... Among the physicians, between the resident and the preceptor....* (P_IU_3)

In addition to the non-participation of non-medical professionals in the IU rounds, the presence of the physician seems, by itself, to establish interprofessional boundaries between professionals and patients.

[doctors] *do not discuss the case. They come and evaluate the patient. If they are there, we move on to the next patient. We don’t stay close....* (E_UNI_6)

As a result, nurses’ participation in bedside rounds is limited due to their heavy workload and the small number of nurses working in the IU.

*We are not used to doing a bedside round with the nursing team. I even think that it would be beneficial... but in the ward it is a little more difficult because the volume of patients is very high and so is the turnover, right? So, sometimes a lot of patients are discharged. The nurses are very busy, so it is very difficult.* (P_IU_5)

*We don’t have a large enough nursing team to make someone available for so long, but from the rest someone always comes: clinical pharmacy, nutrition always has someone, psychology, social service, but from nursing itself, we don’t have anyone*. (KI_P_1)

*Even in the inpatient unit, we have always had low participation from nursing team* [in bedside rounds]. (KI_PSY_1)

Furthermore, the physician quoted below mentions a feeling of ownership of bedside rounds, justifying their decision to do them without the participation of other medical or non-medical professionals.

*So, when I think the bedside round is mine, right?! I do it alone, and I think it’s good that it’s done by myself to build the doctor-patient bond. And I don’t even like that students go with me... I like to feel how the patient is that day, and then it’s my moment with the patient. The preceptor, if he feels like he needs to, goes there after…* (P_IU_4)

On the other hand, nurses also do their rounds alone, reinforcing the fragmentation of care.

[nurses] *do bedside rounds over all the beds. ...so, the nurse in the morning always does all the bedside rounds... Alone. It is her* [the nurse’s] *bedside round alone. There is no multiprofessional round here. It doesn’t exist.* (N_IU_3)

Another characteristic of rounds in the IU is that they do not always take place at the side of patients’ beds. At the nursing station, resident physicians often discuss cases with their preceptors and/or the physician in charge.

*They* [resident and preceptor] *sit at a specific station, go through the cases, and the preceptor doesn’t even go to the patient’s bedside.* (N_IU_1)

*It* [bedside rounds] *doesn’t happen face-to-face. They don’t do bedside rounds. They sit at the computer and discuss the cases. They call it bedside rounding, often taking up the computers of the nursing staff.* (N_IU_4)

*The beside round is only done when it is necessary, only when we think we have some doubt in the patient’s examination, or something like that.* (P_IU_1)

Our data indicates that the lack of standardized routines and norms for conducting rounds impacts the work of the health team both in the ICU and in the IU. Although there are differences between the rounds in the two sectors, the elements and circumstances that influence them are similar. In addition, in both sectors, the physician is central to the organization of the care process.

## DISCUSSION

Data allowed the analysis of the bedside round as a strategy of care that involves the participation of several actors. From a power relations perspective, we can understand it as a tool that shapes a field of knowledge, where the lines of visibility and enunciation regimes facilitate the creation of a specific domain^(10)^.

History is permeated by discursive movements that can be questioned, undone, reconstituted, and replaced. The description of the discursive events serves as a horizon for the formation of units, a manifestation of the subject’s existence, and an expression of his knowledge^(22)^.

According to Foucault, knowledge produces a grounded discourse that sustains a power regime in defense of truth. Thus, it is precisely in discourse that power and knowledge are articulated, whose tactical function is neither uniform nor stable. Knowledge, power, subject, and environment come together in the social context of truth to produce discourses^(12)^.

The bedside round is a procedure that aims to improve inpatient care safety by encouraging interactive, multiprofessional decision-making^(23)^. The bedside round can be considered a form of interdisciplinary interlocution that ensures a fast flow of information related to patient care^(3)^. It is a process that allows the professional team to identify patients’ therapeutic needs and deliver holistic, continuous, and quality care^(15,24)^. It is also a space for reflection on best practices that lead to high-quality health care, where various health care professionals have the opportunity to voice their opinions based on scientific evidence and collectively determine the best patient care strategies^(2)^. The relationship between poor team communication and adverse health events points to the need for structured communication practices among the care team^(25)^.

Structured interprofessional communication, as expected in a bedside round, provides an opportunity for members of the healthcare team to routinely interact for the sake of patient safety and to develop a collaborative therapeutic plan^(25)^. An integrated bedside round requires the full involvement of all team members to guarantee standardized interdisciplinary communication mechanisms that enable scientific discussions to allow collaborative and safe care^(1,15)^.

In any social context, a norm describes the functioning and purpose of a process, promotes synchrony, and standardizes individual conduct in a field of comparisons and rules to follow^(10)^. The existence of a norm suggests a need to discipline the subjects with the purpose of controlling body operations, submitting their forces to what is desired^(9)^. The hospital under study lacks a set schedule or document governing bedside rounds. However, this hospital clearly institutionalizes the rounds, subjecting them to the style of the in-charge physician in both the ICU and the UI.

Due to medical reasons, there is no pre-established schedule for the bedside round, indicating that the presence of non-medical professionals is not considered necessary. This keeps the physician primarily responsible for establishing therapeutic strategies, even those involving other professionals. In this sense, the institutionalization of this physician-centered practice controls the practices of the other professionals who submit to it to perform bedside rounds. It is noteworthy that in the many forms of domination, a certain social group is seen as hegemonic, which establishes power relations with other groups socially seen as subordinate^(10)^.

Despite the potential for positive exchanges between caregivers and patients during bedside rounds, previous research indicates that nurses frequently miss out on these opportunities^(26)^. Moreover, even when they are present in multiprofessional discussions, few nurses effectively participate in or contribute to decision-making^(27,28)^.

Researchers conducted a study in a general hospital in Buenos Aires to understand the dynamics of clinical discussions, yielding results similar to ours. The researchers suggest that physician-centered bedside rounds in this context disregard nurses’ opinions, thereby undermining patient care. This study also found that nurses are usually not even invited to attend other professionals’ conferences^(26)^. Similarly, researchers found that nurses working in an intensive care unit in a Brazilian hospital felt uncomfortable with bedside rounds and experienced anguish for not being involved in decisions that affect patient care^(27,28)^.

In modern medicine, the specialization of professions seeks to ensure quality care for hospitalized patients. On the other hand, this specialization creates very specific fields of knowledge that often constitute barriers to team communication. One strategy to ensure effective communication between members of the care team is to run structured multiprofessional discussions at the bedside^(25)^. But this is still a challenge in the IU, where, unlike in the ICU, our findings suggest that the clinical discussions about the patients do not take place at the bedside. Furthermore, it is common for physicians and nurses to perform their patient evaluations in an isolated and independent way without establishing efficient clinical communication.

Science’s advancement has given rise to a select group of socially authorized voices due to their status as professionals with knowledge that is considered true. Although all professional categories possess and produce knowledge in the field of health, medical discourse has a social value because it is considered the discourse of truth, which has legitimacy, visibility, and scientific recognition^(29)^. Power and its manifestations closely relate to visibility. In human relations, power is ever-present and shifts in response to the shifting positions of the subjects in the dispute for knowledge^(9)^.

Brazilian research suggests that physicians consider themselves responsible for clinical decisions, which gives them greater visibility in power relations by legitimizing their knowledge in discursive practices about patients’ cases, leaving nurses less likely to be seen as holders of knowledge scientific^(30)^.

Similar to our findings, previous research has found that it is common for clinical discussions to establish therapeutic strategies to occur in corridors or rooms and not at the patient’s bedside. We can attribute this to several factors, including time constraints and conflicts in team members’ schedules^(4)^. Although integrated care benefits the patient, some obstacles need to be overcome, including the difficulty of communication between professionals^(31)^.

The participants in our study frequently noted that the participation of nurses in clinical discussions takes them away from administrative routines within the unit, which today are considered indispensable. A study conducted with nurses in Rio Grande do Sul, Brazil, suggested that the administrative and management functions of the sector were associated with work overload, and this resulted in negative impacts on the quality of care because such work removed nurses from their bedside duties^(32)^.

The positioning of professionals in discourses involves power relations that affect people’s daily lives, with language being a vehicle for the manifestation of forces and the exercise of power. A Foucauldian concern is what constitutes the subject through the relationship in which he places and recognizes himself, being that subjectivity is related to games of truth and power established by language, in the search for recognition^(10)^.

### Study limitations

The main limitation of this study is that we examined the relationships and bedside round processes within only one hospital. It is crucial to develop other studies in different settings to more fully understand all the dimensions and effects of various bedside round practices that require different forms of knowledge from different health professionals.

### Contributions to the area of nursing, health or public policy

Given that the effects of this relationship can constitute factors that interfere with the visibility of professionals and the quality of care, this study is an opportunity to collaborate with relevant information for the field of science, health, and nursing, when analyzing how power devices influence in the arrangement of bed runs, to propose improvements in this process.

## FINAL CONSIDERATIONS

This study showed the construction of arguments about the configuration of power relations in bedside rounds. In this sense, the interviewees portrayed bedside rounds as a reflection of the relational environment, and how this aspect depends on the involved professionals’ discourses.

No rules and routines regulate the bedside round process in the hospital: this fact allowed us to perceive that this process is built into daily practices according to power relations established among health professionals. However, the lack of routines and norms that standardize bedside rounds makes collaborative and integrated care difficult both in the IU and in the ICU. Although there are differences between the bedside rounds between the units, care is organized around physicians’ needs in both units.

As power relations aren’t limited to specific human relations but rather present in all of them, we intended to identify nuances of power relations in the interdisciplinary work of health professionals in the bedside rounds process.
